# Switch-like Behavior in the Heme Receptor for *Vibrio Vulnificus*

**DOI:** 10.1007/s11538-025-01505-2

**Published:** 2025-08-09

**Authors:** Kathryn S. Lynch, James P. Keener

**Affiliations:** Mathematics, University of Utah, 155 1400 E, Salt Lake City, 84112 UT USA

**Keywords:** *Vibrio vulnificus*, HupA, Heme, Gene regulatory network, Switch, Saddle node bifurcation

## Abstract

Switch-like behavior and bistability are important features in gene regulatory networks, allowing cells to distinguish between changing environments and express certain genes only under the appropriate conditions. *Vibrio vulnificus*, an opportunistic Gram-negative marine pathogen, has iron as a limiting growth factor. When inside a human host, this bacteria utilizes heme as a source of iron, necessitating the ability to turn this heme acquisition system off and on in response to environmental pressures. As establishment of infection depends on *V. vulnificus*’s ability to change from a marine to human environment, the ability to switch on the heme-intake system is an important part of establishment of initial infection. In particular, the protein HupA is a key part of the bacteria’s heme importation complex, and is regulated primarily by a divergently transcribed protein, HupR. The dynamics of this regulation result in a genetic switch, allowing the bacteria to differentiate between high iron or high heme environments, determining which source of iron should be used. Bifurcation analysis of this network uncovers a saddle-node bifurcation, which encodes this switch-like behavior into the regulation of the heme transport system and allows different levels of expression for HupA depending on external concentrations of heme and iron. The influences of other parameters in this system are also investigated; in particular, promoter leakage is found to be required to enable this bistability, indicating the importance of imperfect regulation in a cell’s ability to respond to the environment.

## Introduction

*Vibrio vulnificus* is an opportunistic Gram-negative marine pathogen, infection by which has an unusually high mortality rate. This halophilic bacteria is typically found in marine environments with relatively warm water temperatures and with lower salinity, such as estuaries (Baker-Austin and Oliver 2018). Although infection by *V. vulnificus *is rare in comparison to other genus members, such as *Vibrio cholerae*, the severity of infection renders it of particular interest. Infection typically occurs in one of two ways: localized infection via contact between an open wound and brackish water or gut infection via consumption of contaminated shellfish. Either method of infection can lead to extreme cases where infection spreads to the bloodstream and causes necrotizing fasciitis or primary septicemia, particularly in patients with underlying conditions (Oliver 2005; Jones and Oliver 2009). In the United States, *V. vulnificus *is responsible for the majority of all seafood related deaths, with the mortality rate estimated as exceeding 50% (Jones and Oliver 2009; Elgaml and Miyoshi 2017). Additionally, infections occur rapidly, with the time between onset of symptoms and clinical outcome in as few as one to two days (Baker-Austin and Oliver 2018).

Despite the severity and speed of infection, the exact virulence factors and mechanism of pathogenicity are not well understood. Although a number of key proteins and toxins have been considered as virulence factors for the disease, their exact role in the virulence of *V. vulnificus *is unclear (Choi and Choi 2022; Jones and Oliver 2009; Miyamoto et al. 2021; Yuan et al. 2020). One study suggests that virulence depends on the exact strain of *V. vulnificus *used (Pipes et al. 2024). Another notes that two of the toxins that have been considered as virulence factors, VvhA and VvpE, are expressed differentially in an infected human depending on the location of infection (Elgaml and Miyoshi 2017). This suggests that virulence and pathogenicity may depend on a complex assortment of factors including environment, location of infection in the human body, and stage of infection (Pipes et al. 2024). A better understanding of the mechanisms by which *V. vulnificus *rapidly establishes itself within a human host could offer an additional explanation for its virulence and suggest potential avenues for treatment.

One possible factor in the establishment of *V. vulnificus *infections is its iron acquisition systems. In *V. vulnificus*, as with other *vibrios*, iron is used in a variety of metabolic pathways and is a growth limiting factor (Miyamoto et al. 2021). However, these iron acquisition pathways must be carefully regulated as an excess of iron can induce iron toxicity, thereby inhibiting bacterial growth (Payne et al. 2016; Runyen-Janecky 2013). Integrating a variety of environmental signals to manage iron acquisition pathways is one of several challenges from a host that bacteria must overcome in order to survive and proliferate. The vast majority of iron in the human body is sequestered in iron binding molecules, with approximately 70% of iron in the human body bound in hemoglobin. Each hemoglobin molecular complex contains four molecules of heme bound to a globin protein, with each heme bound to a single ferrous iron atom. In its oxidized form of hemin, each heme is bound to a ferric iron atom. Bacteria have a variety of ways to acquire iron while within a host; some such as *V. vulnificus *are able to extract iron from the hemoglobin contained in erythrocytes. To this end, these bacteria have a variety of transporters, toxins, and enzymes to extract iron from heme. However, this is an energetically wasteful strategy in iron replete conditions; this, as well as the possibility of excess iron leading to cytotoxycity, means that most of these heme acquisition genes are turned off in iron replete environments (Runyen-Janecky 2013). Expression of these toxins and proteins, as influenced by environmental factors, may be a contributing factor in the virulence of this bacteria.

A key part of the heme-iron acquisition system in *V. vulnificus *is the receptor HupA, an outer membrane receptor for a TonB type transporter responsible for heme importation (Litwin and Byrne 1998). This outer membrane receptor is coupled to a proton motive pump, which enables the transduction of energy across the periplasm and allows for unidirectional transport of the heme-iron complex across the outer membrane once the molecule has bound to the appropriate receptor. The molecule is then bound to a periplasmic binding protein (HupB), delivered to and transported past the inner membrane by ABC transporter HupCD, consisting of a cytoplasmic membrane permease (HupC) and peripheral membrane ATPase (HupD) (Miyamoto et al. 2021; Kawano et al. 2018; Payne et al. 2016). HupA allows for the use of hemoglobin as well as heme; one possible mechanism for this is that HupA, in addition to being a receptor, serves as a protease to liberate the heme-iron complex from hemoglobin (Litwin and Byrne 1998). As the HupA receptor is an essential component of the heme transporter, here we consider only HupA as a representative for the entire transporter.

In this paper, we model the regulation of HupA to better understand the conditions under which the bacteria use heme as a source of iron. We construct a model describing this regulation and analyze the bifurcation structure to glean insight as to how various external signals influence the expression of heme importation genes. Reviewing the model’s response to the extracellular environmental stimuli reveals switch-like behavior. We demonstrate that the model follows appropriate quantitatively described behaviors, to validate that the included regulatory mechanisms are sufficient to explain the *in vitro* observed behaviors. This characteristic behavior may help describe and determine the bacteria’s response to a changing extracellular environment and offer some explanation as to driving mechanisms of the initial infection phase, thereby influencing the virulence of the bacteria.

## Results

### Model Construction

**Fig. 1:**
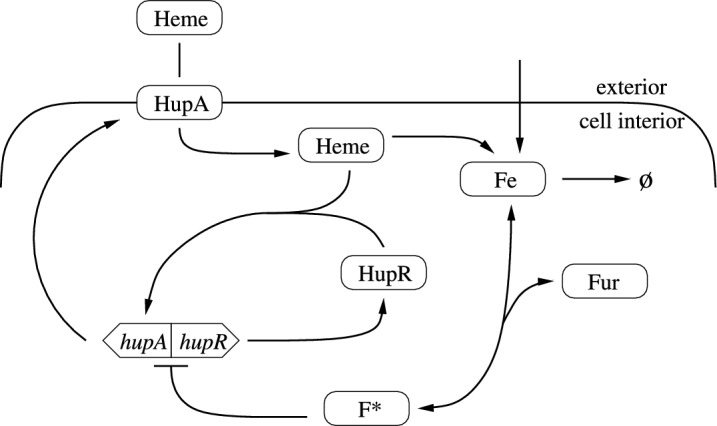
Overview of the HupA regulatory system. The *hupA, hupR* genes produce HupA and HupR, respectively; HupR in turn activates transcription of HupA. Uptake of heme is mediated by HupA; once inside the cell, heme is converted to iron which is in turn used up by the cell for metabolic purposes. Iron is additionally imported from the extracellular environment and binds with Fur to produce transcription factor $F^*$, with this reversible binding indicated with double headed arrows. The flat headed arrow from $F^*$ to the *hupA, hupR* operon indicates repression of both genes

**Fig. 2:**
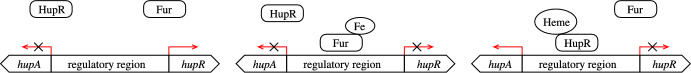
Possible states of the divergently transcribed *hupA* and *hupR* operon, where the direction of transcription is controlled by transcription factor binding to the shared regulatory region. Each transcription factor (HupR and Fur) is activated in the presence of heme or iron, respectively and is then able to bind to the regulatory region. Red arrows indicate the direction of transcription and X’s indicate transcription is repressed in that direction. When nothing is bound, HupR is transcribed and HupA is repressed; when Fur is bound, nothing is produced; and when HupR is bound, HupA is transcribed and HupR is repressed

To model the expression of HupA, we examine the relevant gene regulatory network, seen in Figure 1. *hupA* is located upstream and in the opposite direction of *hupR*, which encodes a transcription factor (Kawano et al. 2018). These genes are divergently transcribed, meaning they share a regulatory region and are transcribed in opposite directions starting from the promoter binding sites in this region. The direction of transcription is determined by the binding of various transcription factors within the regulatory region, as seen in Figure 2. In this instance, two transcription factors affect the operon state: HupR (*R*) and Fur (*F*). When HupR (*R*) in the presence of heme (*H*) is bound (operon state $O_R$), HupA is transcribed, when activated Fur ($F^*$) is bound (operon state $O_F$), both genes are repressed, and when nothing is bound (operon state $O_0$), HupR is constitutively produced. Additionally, the binding regions for both of these transcription factors are relatively close together in the sequencing of the shared regulatory region (Litwin and Byrne 1998; Litwin and Quackenbush 2001). As such, we assume that Fur and HupR cannot be simultaneously bound, and the operon only exhibits the three states depicted in Figure 2. The transition between these states is then

$$\begin{aligned} O_R \begin{array}{c} \scriptscriptstyle k_{r-}\\ \longrightarrow \\ \longleftarrow \\ \scriptscriptstyle k_r \end{array} O_0 \begin{array}{c} \scriptscriptstyle k_f\\ \longrightarrow \\ \longleftarrow \\ \scriptscriptstyle k_{f-} \end{array} O_F, \end{aligned}$$

where the transition between these states is mediated by the binding and unbinding of transcription factors. HupR is a LysR type transcription factor activated in the presence of heme (*H*) (Litwin and Quackenbush 2001). These transcription factors typically have strong dimerization, activation by a ligand, and often interact with DNA at multiple binding sites. The classic configuration involves two transcription factor dimers, each activated by a single coinducer interacting with three DNA regions. This dimer of dimers activates transcription via a “sliding dimer” mechanism (Demeester et al. 2024). Since these interactions seem to happen only in the presence of DNA, we do not consider a separate activated transcription factor. Instead, let the transition from $O_0$ to $O_R$ depend on the simultaneous interaction of *n* dimers of HupR, each activated by a single molecule of heme. The regulatory region has the LysR characteristic symmetrical guanine bases in the two dyad arms of the *hupA* *hupR* shared regulatory region; unlike the most classic LysR transcription factor, this involves two DNA interaction sites (Litwin and Quackenbush 2001). However, less is known about these nonclassical LysR type transcription factors. To account for this, we consider both the single dimer ($n=1$) and classic dimer of dimers ($n=2$) cases.

Then transcription of HupR and HupA can be described as a rate dependent on the probability of the operon being in the relevant state. Suppose $P_R$ is the probability of having HupR (*R*) and heme (*H*) bound, in which case HupA is transcribed, $P_F$ the probability Fur ($F^*$) is bound, and $P_0$ the probability that nothing is bound, in which case HupR is transcribed. Then the probability of the operon being in these three states is described by the following equations:

$$\begin{aligned} \dot{P_R}&= k_r R^{2n} H^n P_0 - k_{r-} P_R, \\ \dot{P_F}&= k_f F^* P_0 - k_{f-} P_F, \\ \dot{P_0}&a= 1 - P_R - P_F, \end{aligned}$$

We assume that these binding rates are fast relative to other processes in the system and put the equations in steady state. This gives us steady state probabilities

$$\begin{aligned} P_R = \frac{b_1 R^{2n} H^n}{b_1 R^{2n} H^n + b_2 F^* +1 }, &  P_0 = \frac{1}{b_2 R^{2n} H^n + b_2 F^* + 1 }, \end{aligned}$$

of the operon being bound by HupR or unbound, where $b_1 = \frac{k_r}{k_{r-}}$ and $b_2 = \frac{k_f}{k_{f-}}$.

We use the diagram in Figure 1 to model the protein dynamics. As this portion of the heme acquisition system does not rely on post-transcriptional regulation, we assume that the process of translation occurs at a relatively fast rate, allowing us to consider both transcription and translation as a single process. Then HupA is created at a rate directly proportional to the probability that the operon is bound by HupR ($P_R$) and degrades at a rate proportional to its concentration (*A*). We make the same assumptions for HupR. Of course, gene regulation is imperfect and there is a small amount of leakage between all three operon states. In this model, we include leakage ($\epsilon$) in the state $O_0$, which corresponds to a small probability of transcribing HupA even when HupR is not bound to the DNA. The natural stochasticity of RNA polymerase binding to DNA ensures that small amounts of HupA or HupR will be produced even when the operon is not in the correct state; taking a mean field approximation ensures that in most cases the deterministic model is sufficient. However, in the case of HupA, some amount of heme is required for the expression of HupA, and heme in turn must be imported by HupA. Since this makes it possible for the model to get stuck in a zero HupA steady state, we include a leak term to account for this stochastically driven, low level production of HupA. Then the concentrations of HupA (*A*) and HupR (*R*) can be described by the following equations:

1 $$\begin{aligned} \dot{A}&= a_1 P_R + \epsilon - d_1A = a_1 \frac{b_1 R^{2n} H^n}{b_1 R^{2n} H^n + b_2 F^* + 1 } + \epsilon - d_1 A, \end{aligned}$$

2 $$\begin{aligned} \dot{R}&= a_2 P_0 - d_2 R = a_2 \frac{1}{b_2 R^{2n} H^n + b_2 F^* + 1 } - d_2 R. \end{aligned}$$

Given some nonzero external concentration of heme ($H_{ext}$), heme is transported into the cell following Michaelis-Menten or saturating kinetics, $V(H_{ext}) = \frac{H_{ext}}{K_H+H_{ext}}$. Once inside the cell, heme is broken down to extract the bound iron, likely following an enzymatic process similar to what is seen in other *vibrios* (Miyamoto et al. 2021; Runyen-Janecky 2013). In this case, since iron is required for metabolism, the reaction should have a high binding affinity and fast velocity to ensure the bacteria does not enter iron starvation. We assume the reaction is fast enough so that it never saturates and can be approximated as a linear term. The corresponding equation for the concentration of intracellular heme (*H*) is then

3 $$\begin{aligned} \dot{H } = a_3 V(H_{ext}) A - a_4 H. \end{aligned}$$

Once extracted from heme, iron (*Fe*) is used for various regulatory and metabolic purposes within the cell. We account for all usage and degradation of iron with a single linear term, so that iron is eliminated from the system with rate $d_3$. As well as these metabolic pathways, iron binds to and activates transcription factor Fur (*F*); this binding and unbinding, with rates $k_1$ and $k_{1-}$, respectively, follows the law of mass action. Master regulator Fur is conserved across many species of *Vibrios* and acts as a suppressor of iron acquisition pathways (Payne et al. 2016; Miyamoto et al. 2021). This transcription factor represses many genes in iron acquisition pathways to prevent toxicity from iron overload (Richard et al. 2019). As the creation and degradation of Fur is not affected by heme or other factors in this model, we assume that the amount of Fur in the cell is conserved, so that $F+F^*=F_{T}$ for some total concentration of Fur, $F_T$. The equations for the concentration of intracellular iron (*Fe*) and activated Fur ($F^*$) are then

4 $$\begin{aligned} \dot{Fe}&= a_4 H - k_1 F Fe + k_{1-}F^* - d_3 Fe +G(F_{ext},F^*), \end{aligned}$$

5 $$\begin{aligned} \dot{F^*}&= k_1 F Fe - k_{1-} F^*, \end{aligned}$$

where $G(F_{ext},F^*)$ describes the importation of iron from non-heme sources, allowing us to gain a complete picture of the systems response to varying levels of external nutrient sources. This transport is assumed to be unidirectional and follow Michaelis-Menten kinetics with a nonspecific mechanism for iron intake. We assume that, as with other iron acquisition pathways, this transport is repressed by activated Fur ($F^*$) under iron replete conditions. This gives the function describing usage of external iron not bound to heme

$$\begin{aligned} G(Fe, F^*) = k_2 \frac{1}{k_3 F^* + 1} \frac{F_{ext}}{K_{F} + F_{ext}} \end{aligned}$$

### Nondimensionalization

We nondimensionalize equations 1 - 5 according to the values in Table 2, where the lowercase *a*, *r*, *h*, *fe*, and $f^*$ correspond to the nondimensionalized values of *A*, *R*, *H*, *Fe*, and $F^*$, respectively. Putting all of this together, the full system of equations modeling the expression of HupA is

6 $$\begin{aligned} \dot{a}&= \alpha _1 \left( \frac{\beta _1 r^{2n} h^{n}}{\beta _1 r^{2n} h^{n} + \beta _2 f^* + 1} + \epsilon - a \right) , \end{aligned}$$

7 $$\begin{aligned} \dot{r}&= \alpha _2 \left( \frac{1}{\beta _1 r^{2n} h^{n} + \beta _2 f^* + 1} - r\right) , \end{aligned}$$

8 $$\begin{aligned} \dot{h}&= \alpha _3 V(h_{ext}) a - h, \end{aligned}$$

9 $$\begin{aligned} \dot{fe}&= \alpha _4 h - \alpha _{4-} fe + \kappa _2 \frac{1}{1+\kappa _3 f^*} \frac{f_{ext}}{1 + f_{ext}} - \kappa _1 \left( (f_T - f^*) fe - f^* \right) , \end{aligned}$$

10 $$\begin{aligned} \dot{f^*}&= \kappa _1 \left( (f_T - f^*) fe - f^* \right) . \end{aligned}$$

### Simplified Model: Constant $f^*$

We initially consider a reduced model, which can be found by knocking out the transition between heme and iron ($\alpha _4=0$) so that we consider only equations 6 - 8. Then $f^*$ can be assumed to be a parameter, and we determine if the regulatory dynamics of the *hupA / hupR* operon result in switch-like dynamics for a constant value of $f^*$.

Consider the case where $n=1$, corresponding to a single HupR dimer interacting with a single molecule of heme. We solve equations 6 and 7 using the resultant as in (Keener 2025). Eliminating *r*, we find

11 $$\begin{aligned} 0 = h \left( a-\epsilon -1\right) ^{3} \beta _1+\left( \beta _2 f^*+1\right) ^{3} \left( a-\epsilon \right) , \end{aligned}$$

at steady state. We then solve equation 8 for $h =\alpha _3 V(h_{ext}) a$ and plug this into equation 11. We can then implicitly solve for HupA as a function of external heme, finding

12 $$\begin{aligned} V(h_{ext}) = -\frac{ \left( \beta _2 f^*+1\right) ^{3} \left( a-\epsilon \right) }{a \alpha _3 \beta _1 \left( a-\epsilon -1\right) ^{3}} . \end{aligned}$$

If this function is cubic in nature, it is possible that there is hysteretic behavior. We can check this by looking for critical points of the derivative of equation 12 with respect to the variable of interest, *a*, where we restrict *a* to be positive, as is biologically feasible. Then a saddle node will occur at zeros of

$$\begin{aligned} \frac{d V(h_{ext})}{da} = \frac{ \left( 3 a^{2}-4 a \epsilon +\epsilon ^{2}+\epsilon \right) \left( \beta _2 f^*+1\right) ^{3}}{\left( a-\epsilon -1\right) ^{4} \alpha _3 \,a^{2} \beta _1} . \end{aligned}$$

**Fig. 3:**
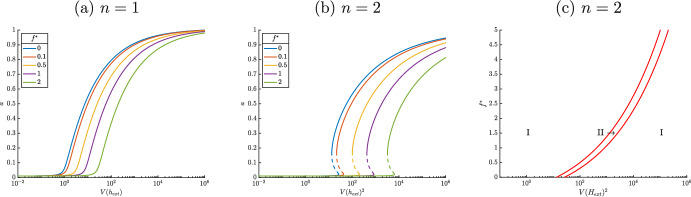
Steady state as a function of external heme for the reduced model (equations 6 - 8). Other parameters are set as $n=1$, $\beta _1=1$, $\beta _2=1$, $\alpha _3=1$, and $\epsilon =0.01$. Location of saddle nodes for equations 6 - 8 as a function of $f^*$ and $V(h_{ext})^2$. In region I, the system has a single steady state solution and in region II the system is bistable. HupA is expressed at a low level to the left of the bistable region and at a high level to the right of the bistable region. Parameters are set as $n=2$, $\beta _1=1$, $\beta _2=1$, $\alpha _3=1$, and $\epsilon =0.01$

It follows that a saddle node occurs if $3a^2 - 4a\epsilon + \epsilon ^2 + \epsilon$ changes signs. The discriminant of this polynomial is $4\epsilon ^2 - 12\epsilon$ which is always negative, since $\epsilon$ is assumed to be small, corresponding to a monotonic steady state curve in *a* and *V*(*h*). These results are seen in Figure 3, where expression of HupA is responsive to increasing levels of heme but does not display switch like behavior. The level of HupA is low for low values of $V(h_{ext})$; after some threshold, *a* increases as $V(h_{ext})$ increases, before again leveling off. This monotonic response is damped by increased $f^*$, with higher levels of $f^*$ requiring a greater amount of $V(h_{ext})$ to activate HupA, but even for high levels of $f^*$ the response curve has the same long term behavior; for high enough values of external heme, HupA will be expressed. Overall, the system has only a single steady state when the activating transcription factor is a single heme dimer ($r^2\varvec{h}$); although expression of HupA is sensitive to $V(h_{ext})$, a single dimer of a LysR type transcription factor is thus insufficient to generate switch like behavior.

By contrast, consider the case $n=2$ corresponding to a more typical LysR type transcription factor with two transcription factor dimers each with a single activating ligands. We can follow largely the same procedure as above, and find that at steady state

$$\begin{aligned} V(h_{ext})^2 = \frac{ \left( \beta _2 f^*+1\right) ^{5} \left( a-\epsilon \right) }{\beta _1 \,\alpha _3^{2} a^{2} \left( a-\epsilon -1\right) ^{5}}. \end{aligned}$$

As in the $n=1$ case, a saddle node bifurcation in $V(h_{ext})$ occurs at a local maximum or minimum of the above equation. By examining

13 $$\begin{aligned} \frac{d V(h_{ext})^2}{da} = \frac{\left( 6 a^{2}-8 a \epsilon +2 \epsilon ^{2}-a+2 \epsilon \right) \left( \beta _2 f^*+1\right) ^{5} }{\left( a-\epsilon -1\right) ^{6} a^{3} \alpha _3^{2} \beta _1} , \end{aligned}$$

we see that saddle node bifurcations occur at zeros of $\left( 6 a^{2}-8 a \epsilon +2 \epsilon ^{2}-a+2 \epsilon \right)$. The discriminant of this is $16\epsilon ^2-32\epsilon +1$, which is positive for small $\epsilon$, corresponding to two roots of equation 13 and two saddle node bifurcations. In Figure 3, it is seen that this is the case. For low levels of $V(h_{ext})$, *a* is low and for high levels of $V(h_{ext})$, *a* is high, with a bistable region mediating the transition between them unlike the monotonic curve in Figure 3. Due to the bistable region in between the saddle nodes, the amount of external heme where the switch in expression of HupA occurs is different depending on whether external heme is increasing or decreasing. This hysteretic behavior defines a genetic switch where, for changing levels of heme, expression of HupA will turn on or off at the saddle nodes, depending on the direction of this change. Additionally, in Figure 3 the steady state curve moves to the right as $f^*$ increases. We see that the amount of $f^*$, or strength of inhibition in the system, increases the amount of $V(h_{ext})$ required to activate expression of HupA. Examining this more detail the influence of $f^*$ on the saddle nodes, we find the location of the cusp in $f^*$ and $V(h_{ext})^2$ which occurs when the two saddle nodes collide, or at a simultaneous zero of equation 13 and its derivative with respect to *a*. We find this again using the resultant and plot the resulting location of saddle nodes in Figure 3. Here the cusp is located in the negative region, which is biologically unattainable. Although larger amounts of $V(h_{ext})$ are required to drive the system into the rightmost high HupA region for larger $f^*$, it is apparent that the saddle nodes persist even for high levels of repression. This is an artifact of the reduced model that a more sensitive repression by $f^*$ will account for in the full model, as increasing amounts of heme result in increased $f^*$.

Overall, from this simplified model we find that the positive feedback coupled with divergent transcription drives switch-like behavior. Other biologically unrealistic cases, such as unlinking the powers of *r* and *h*, also admit hysteresis, but for the realistic cases considered, a second dimeric transcription factor ($n=2$), as mirrors the classic LysR type, is necessary to generate a genetic switch. Although repression via $f^*$ damps the response of *a* to $h_{ext}$, the switch persists for all values of $f^*$, indicating the necessity of including the transition of heme to iron and multiple iron sources in the full model to allow the system to more intelligently decide when to turn on and off HupA. Examining the bifurcation structure of the full biological model will allow us to determine in more detail how the parameters in the system drive this switch like behavior.

### Homotopy

Now that we have the bifurcation structure for the simplified model, we use this to perform a numerical bifurcation analysis of the full biological system. We do so by defining a homotopy mapping between the reduced (equations 6 - 8) and full (equations 6 - 10) systems. This homotopy defines a continuous transition between the two models, such that $\theta =0$ corresponds to the reduced model and $\theta =1$ to the full biologically relevant model. Then examining the effects of this perturbation in the interval $\theta \in [0,1]$ allows us to intelligently tune the scaling parameters so that the bifurcation structure is preserved across the two models.

$$\begin{aligned} \frac{d \vec {homotopy}}{dt} = (1-\theta ) \frac{d \vec {reduced}}{dt} + \theta \frac{d \vec {full}}{dt} \end{aligned}$$

**Fig. 4:**
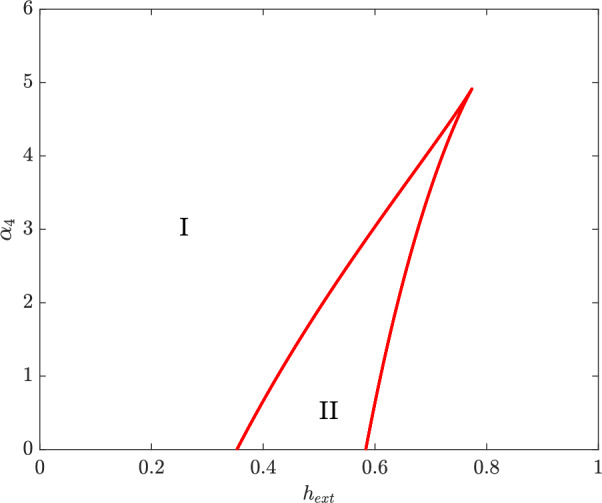
Location of saddle nodes in equations 6 - 10. In region I, the system is monostable and in region II bistable. The *x*-axis ($\alpha _4=0$) corresponds to the simplified system, equations 6 - 8. Parameters as in Table 1

To transition between models, we set $V(h_{ext}) = \frac{h_{ext}}{1+h_{ext}}$, to account for a more realistic importation of heme, and set $\alpha _4=\theta$ as the homotopy parameter, gradually reintroducing the conversion of heme into iron. This completes the negative feedback loop in the system, enabling a more sensitive regulation of HupA via iron-activated Fur. In Figure 4, we see how external heme and $\alpha _4$ determine the location of the saddle nodes. A horizontal slice of this graph tells us where saddle nodes occur as a function of $h_{ext}$. Taking a horizontal slice of the graph at $\alpha _4=0$ corresponds to the reduced model, as seen in Figure 3, with two saddle nodes present for all values of $f^*$. Moving upwards, we find that the two saddle nodes persist until roughly $\alpha _4=5$. Although high enough $\alpha _4$ results in a purely monostable system, above the cusp, the persistence of saddle nodes between $\alpha _4=0$ and 1 is sufficient to indicate that the hysteretic structure found in Figure 3 is conserved in the full biological model.

### Bistability in the Biological Model

**Fig. 5:**
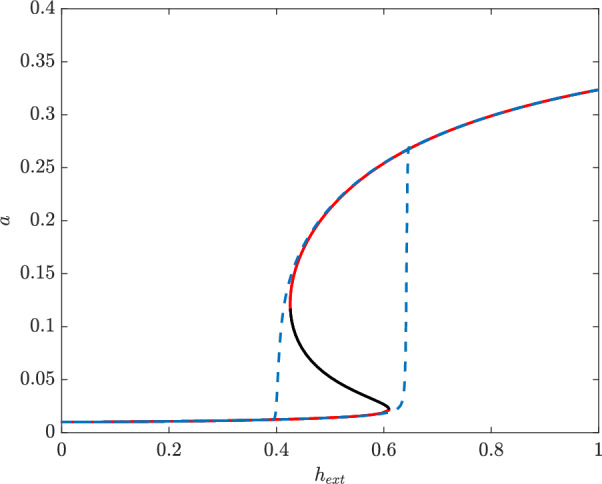
Steady state solution of HupA as a function of HupA, for the parameters listed in Table 2. The red curve represents a stable steady state and black unstable. The dashed line indicates a trajectory of the system simulated for varying levels of external heme. The system contains two saddle nodes, corresponding to a two-way genetic switch in HupA as a function of heme. The hysteresis ensures that production of HupA turns off at a higher level (the left saddle node) than it turns off (the right saddle node)

Considering equations 6 - 10, the two saddle nodes seen in Figure 3 are conserved and can be seen in Figure 5. This hysteretic curve allows the system to function as a two-way biological switch and respond to variable environmental cues. For low levels of heme, *hupA* is off and only a small constitutive amount is produced. For high levels of external heme, *hupA* is on and a much larger amount of HupA is present. In between these two regions, the system exhibits bistability with the long term state of the cell (i.e. the amount of HupA produced) depending on the initial concentration of external heme.

**Fig. 6:**
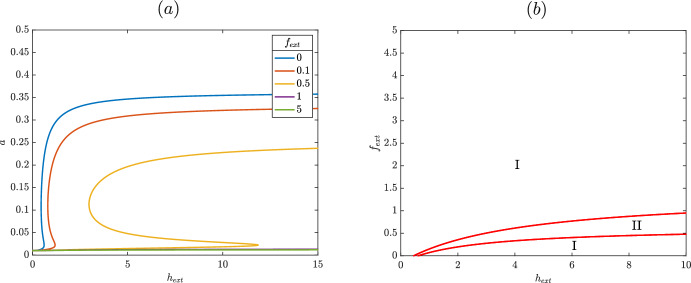
Location of saddle nodes depending on external heme and external iron, with $\beta _1=4$. In region I, the system has a single stable steady state. In region II, the system is bistable. Steady state solutions as a function of $h_{ext}$ for varying amounts of $fe_{ext}$. The solid curves denote a stable steady state and dashed curve an unstable one. Other parameters listed in Table 2

In addition to expressing HupA in the presence of heme, this model is accurate only if it does not express a high level HupA in iron replete conditions. This behavior has been seen in various *in vitro* experiments, where iron represses expression of HupA even in the presence of heme (Litwin and Byrne 1998; Litwin and Quackenbush 2001; Jones and Oliver 2009). Although the exact values of parameters in this model are unknown, tuning parameters so that the model expresses this qualitative data helps validate the model and confirms that these regulatory aspects are the driving forces behind the observed behavior. In Figure 6, we can see the influence of both heme and iron on the location of this switch for two different values of $\beta _1$. The cusp point for these two parameters in both cases is in the negative region and thus biologically unattainable. In both cases, with a low level of external iron, the expression of HupA depends on the concentration of external heme. For low heme, HupA is not expressed except at the very low constitutive level; as the concentration of external heme increases, the solution moves into the bistable region and crosses into the rightmost monostable region, where HupA is highly expressed. Likewise, in both cases, HupA is not expressed in high iron high heme scenarios. In Figure 6(b), when $\beta _1=2$, the upper monostable region corresponds to a low expression of HupA and the lower to high expression of HupA. For low $f_{ext}$, increasing amounts of eternal heme turns on expression of HupA, as seen in Figure 5. However, both branches of the saddle nodes tend towards infinite $h_{ext}$ for finite $f_{ext}$. This means that the monostable “on” state is entirely inaccessible for high iron, even for high concentrations of heme. We see this in Figure 6(a), where expression of HupA can be turned on in a low iron environment. As in the reduced model, increasing the amount of external heme in a low iron setting informs the cell of this alternative iron availability and prompts it to turn on expression of heme acquisition pathways such as HupA. However, unlike the reduced model in Figure 3, HupA in Figure 6 i s suppressed for high levels of iron, seen in the flat curves. These additional details of repression included in the full model allow for a more intelligent decision of when to express HupA, matching *in vitro* observations. Hysteresis does not persist in high iron environments, corresponding well to *V. vulnificus*’s preference for extracellular iron even in the presence of heme.

**Fig. 7:**
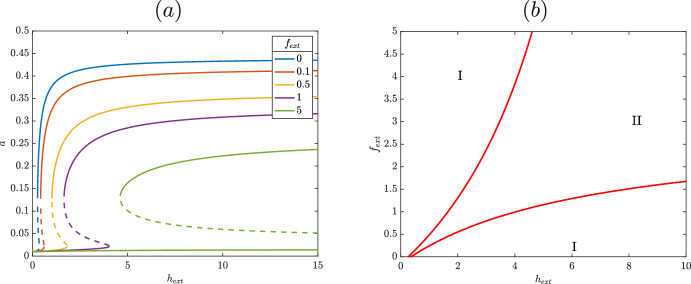
Location of saddle nodes depending on external heme and external iron, with $\beta _1=4$. In region I, the system has a single stable steady state. In region II, the system is bistable. Steady state solutions as a function of $h_{ext}$ for varying amounts of $fe_{ext}$. The solid curves denote a stable steady state and dashed curve an unstable one. Other parameters listed in Table 2

This preference is also present when $\beta _1=4$, as seen in Figures 7. As when $\beta _1=0.7$, the cusp in $h_{ext}$ and $f_{ext}$ is again inaccessible and in the negative region of the graph in Figure 7(b). The upper monostable region corresponds to low expression of HupA and the lower to high expression, with a bistable region in the middle. For low iron, the system when $\beta _1=4$ functions identically to the $\beta _1=2$ case, with increasing heme activating expression of HupA as the system moves from the low monostable region to the high, corresponding to the hyeteretic curves. As in the $\beta _1=2$ case, the lower saddle node tends towards infinity in $h_{ext}$ for finite $f_{ext}$; however, here the upper saddle node tends towards infinite $f_{ext}$ for finite $h_{ext}$. When $f_{ext}$ is high for $\beta _1=4$, the bistable region is still accessible although the monostable “on” region is not. In Figure 7, the suppression of HupA expression for high iron is seen not in a flat curve but in a solution curve with one saddle node bifurcation. For high levels of heme, the system always maintains bistability, with both a high and low HupA steady state solution possible. However, the single saddle nodes makes this a one-way switch; once heme is decreased so that expression of HupA turns off, it will not turn back on even once levels of heme are increased again. This still generally matches the need for *V. vulnificus *to not turn on HupA in a high iron high heme environment, as bacteria moved from a high iron low heme environment to a high iron high heme environment will still not express HupA. However, this suggests that with increased $\beta _1$, corresponding to a stronger affinity between HupR and the binding site, it may be possible to observe high levels of HupA expression in such an environment. The natural stochasticity in gene regulatory networks ensures that a small fraction of *V. vulnificus *in this bistable region will express HupA. Additionally, solutions in the bistable region depend on the initial condition or previous environment; *hupA* will not switch on when moved from a low heme to high heme environment so long as iron concentration remains high. However, if the bacteria starts in a high heme low iron environment and moves to a high heme high iron environment, this model predicts that the bacteria will continue to produce HupA. Here we expect, unlike in the stochastic case, the majority of a colony to continue producing HupA even though heme should not be the preferred source of iron. This indicates the importance of memory in this gene regulatory network; this network retains some sense of past environment to inform its present state. Both cases fit the observed preference for iron over heme when possible, as seen in (Litwin and Quackenbush 2001) ; testing to see whether *V. vulnificus *expresses heme under a high-iron high-heme environment following a low-iron high-heme environment would provide important information about the relative binding affinity of HupA and Fur. If this expression does not happen (i.e. the bistable region is inaccessible for high iron) then the binding affinity of activated HupR must be stronger relative to the other case.

**Fig. 8:**
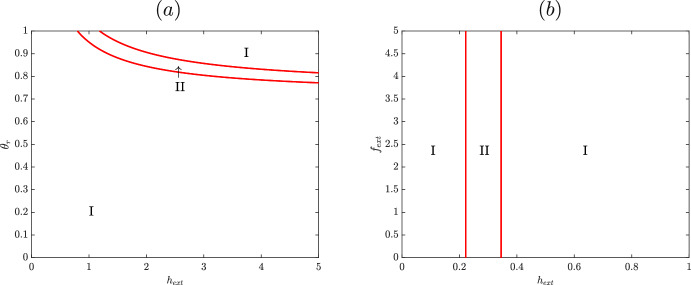
Location of saddle nodes depending on external heme and (a) scaling of HupR creation or (b) external iron. In region I, the system has a single stable steady state. In region II, the system is bistable. In the lower region I, only the lower steady state is accessible and in the upper region I only the higher steady state is accessible. Parameters listed in Table 2, with $f_T=0$ for (b)

To further validate our model, we replicate an experiment by Kawano et. al., who determine the effect of HupR on HupA production by creating a $\Delta$*hupR* mutant; ultimately, they find that $\Delta$*hupR* mutant has significantly reduced production of HupA as compared to the wildtype (Kawano et al. 2018). We replicate this experiment by knocking out the model’s ability to produce HupR. To do so, take Equation 7, which describes the concentration of HupR,

$$\begin{aligned} \dot{r} = \alpha _2 \left( \frac{1}{\beta _1 r^{2n} h^{n} + \beta _2 f^* + 1} - r\right) , \end{aligned}$$

and replace it with

14 $$\begin{aligned} \dot{r} = \alpha _2 \left( \theta _r \frac{1}{\beta _1 r^{2n} h^{n} + \beta _2 f^* + 1} - r\right) , \end{aligned}$$

where $\theta _r$ ranges from 0 to 1. At $\theta _r=1$, Equations 7 and 14 are identical, analogous to a wildtype bacteria. In this case, the above model (Equations 6 - 10) and the previous results hold, as in Figures 5, 6, and 7. At $\theta _r=0$, the equation describing the concentration of HupR in the model becomes $\dot{r} = -\alpha _2 r$ so that the model cannot produce any HupR and the only steady state is $r=0$; this corresponds to a $\Delta$*hupR* mutant. In Figure 8(a), the two parameter bifurcation diagram shows the results of this knockout. At the *x*-axis of the graph, $\theta _r=0$, we see that the two saddle node bifurcations and the high-steady state only region are inaccessible; without the activating influence of HupR, a lower amount of HupA will be produced even in a high heme low iron environment, as in (Kawano et al. 2018). Intermediary values of $\theta _r$ to an partial knockdown of the gene, or one where the cell’s ability to make HupR is significantly reduced but not abrogated. In this instance, it may be possible for a bacterium to produce sufficient HupR to activate HuPA, depending on the severity of the knockdown. However, the wide range of $\theta _r$ values for which the saddle nodes are inaccessible confirms that even an imperfect knockout of HupR is likely to significantly reduce a bacterium’s ability to produce HupA.

Kawano et. al. additionally verify the effect of Fur on HupA by knocking out *fur* to confirm that it is necessary for the repression of HupA in a high iron environment. We replicate this experiment by taking the original model (Equations 6 - 10) and setting $f_T=0$; with the total amount of Fur in the system equal to 0, the model now describes a $\Delta$*fur* mutant. The results of this are plotted in Figure 8(b), where we see that without Fur, external iron has no effect on the location of the saddle nodes; in a high heme environment, HupA will be expressed regardless of Fur. This mirrors the results from the knockout experiments, further validating the model.

### Influence of Other Parameters

**Fig. 9:**
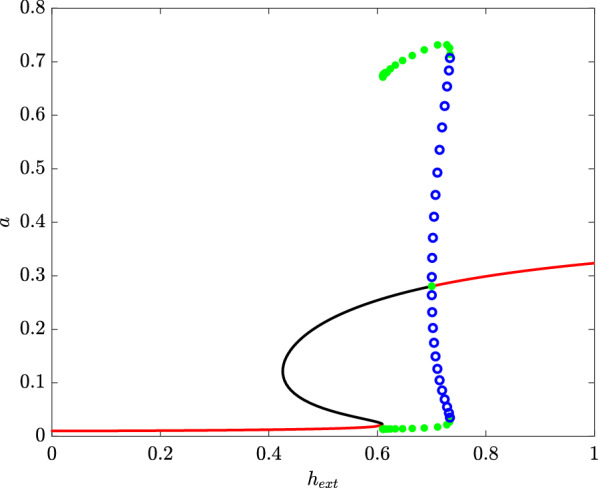
Steady state solution of HupA as a function of external heme, for the parameters listed in Table 2 but with $\alpha _2=0.01$. As with Figure 5, the red curve represents a stable steady state and black unstable. The system contains two saddle nodes, corresponding to a two-way genetic switch in HupA as a function of heme. The system undergoes a subcritical Hopf bifurcation with unstable periodic orbits (blue circles) emanating from the bifurcation point. A saddle node periodic bifurcation delineates the transition from unstable to stable periodic orbits (green circles). These stable periodic orbits terminate in a homoclinic orbit, which occurs very close to the lower saddle node

We also consider the effects of other parameters on the bifurcation structure, which inform the existence and location of hysteresis. By numerically finding the location of the cusp in external heme and various other parameters, we gain insight into how the tuning of this network informs its functionality.

First we consider the time scaling parameters $\alpha _1$ and $\alpha _2$, which correspond to the nondimensionalized maximal production rates of HupA and HupR, respectively. We find that changing these parameters does not affect the location of hysteresis; however, a Hopf bifurcation may emerge, seen in Figure 9. The hysteretic curve here is identical to that seen in Figure 5, but with a subcritical Hopf bifurcation on the upper stable branch. These unstable oscillations pass through a saddle node periodic (SNP) bifurcation and change stability before terminating in a homoclinic bifurcation near the rightmost saddle node. Within this region, concentration of HupA would oscillate between high and low; close to the homoclinic bifurcation, the system would spend the majority of its time in the low HupA state. From this, we can see that if the ratio $\alpha _1/\alpha _2$ is large, then the system still exhibits the same hysteretic behavior but with oscillatory solutions along the upper stable branch. This is a relevant parameter regime only in the instance that the maximal production rate of HupR is much slower than the production rate of HupA. While mathematically possible, it is unlikely that there are oscillations in this system and so we predict the maximal production rates of HupA and HupR to be relatively close.

**Fig. 10:**
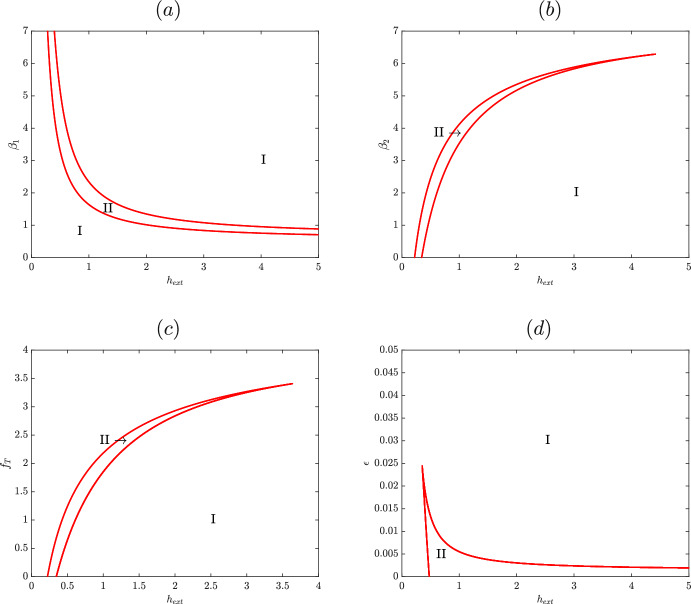
Location of saddle nodes depending on external heme and binding affinity of activated HupR. In region I, the system has a single stable steady state. In region II, the system is bistable. Parameters listed in Table C

Next we consider the binding affinities of HupR and activated Fur ($\beta _1$ and $\beta _2$, respectively) with the regulatory region. In gene regulatory networks, the binding affinities of various transcription factors often play a key part in determining the functionality of the network; as such, genetic switches often rely on correct tuning of these binding affinities. In Figure 10, saddle nodes are close together for high values of $\beta _1$. From this, we see that a high binding affinity between HupR and the operon results in a switch that activates even for low values of external heme. Additionally, this switch will flip on and off relatively quickly, due to small bistable region. However, for lower $\beta _1$, the saddle nodes are increasingly further apart and, for sufficiently low $\beta _1$, neither saddle node is accessible. This corresponds to a low binding affinity between HupR and DNA; in such a regime, HupA is never able to turn on due to the ineffectiveness of transcription factor HupR. In Figure 10, on the other hand, the cusp point occurs for high $\beta _2$ and high heme. For decreasing $\beta _2$, the saddle nodes are further apart and occur at lower levels of external heme. As a result, the switch is preserved even with low affinity between activated Fur ($f^*$) and the binding region. If $\beta _2$ is too high, however, repression by $f^*$ is too strong and HupA is not able to turn on; if this case is more accurate, HupA will exhibit a sensitivity to external heme concentration but will not display hysteresis. This careful balancing of binding affinities is important in the structure of a gene regulatory network; in this case, these affinities can be tuned to elucidate a switch or sensitivity.

Figure 10 shows how the overall availability of Fur (including both inactive and activated) informs the system. The cusp here occurs for high $f_T$ and high $h_{ext}$ and decreasing $f_T$ decreases the value of heme for which saddle nodes occur. As the total amount of Fur increases, increasing amounts of heme are required to turn on expression of HupA. Eventually, the amount of Fur increases so much that HupA can never turn on. In this scenario, there is so much repressive transcription factor present that both HupA and HupR are repressed regardless of the nutrient availability in the environment. While the downregulation provided by Fur is essential in protecting the cell from iron toxicity and in ensuring that HupA is not turned on in high iron environments, the amounts of this transcription factor must be carefully balanced to ensure HupA is still able to turn on.

Finally, we consider the two parameter bifurcation diagram in external heme and leakage, $\epsilon$, in Figure 10. In this case, bistability is only possible for low values of $\epsilon$ and heme, with the cusp appearing near $\epsilon =0.025$. The left saddle node in the system stays relatively constant for decreasing values of $\epsilon$, but the right saddle node moves towards infinity as $\epsilon$ decreases. For high values of $\epsilon$, the system remains in a monostable state even as the amount of external heme increases. While in this region the bacteria can still respond to environmental input, it cannot sharply adapt to changes. This high leakage corresponds to an unrealistically high level of constitutive HupA expression. At the other extreme, when there is no leakage, $\epsilon =0$, increasing heme allows the system to cross one limit point, entering the bistable region, but not the second due to the low asymptote of the rightmost branch of the cusp. In this case, the bacteria has no ability to turn on its heme importation system, even in environments where this would be preferred. A mechanism for sensing heme in the environment such as this small but nonzero constitutive expression of HupA, even in high iron environments is required for the bacteria to switch on expression of heme acquisition genes only in the presence of heme. A similar small amount of leakage happens for every gene regulation action in this model; however, not including other leakage terms does not affect the functionality of the model. This demonstrates the importance of leakage to the functionality of an activating switch. Leakage is not simply an artifact of imperfect regulation and stochasticity, but also a necessary part in this regulatory network’s ability to function as a switch.

## Discussion

In this paper, we determined the bifurcation structure for a complex gene regulatory network governing the expression of a heme receptor. This approach to analyzing a complex gene regulatory network demonstrates a method to make use of essential regulatory features of other similarly complex networks to understand the effect of their structure.

While this model makes use of qualitative *in vitro* observations of this regulatory network, further *in vitro* confirmation of these predictions would further validate the model’s accuracy. In particular, it is known that *V. vulnificus *produces larger amounts of HupA only in a heme-rich, iron-replete environment (Litwin and Byrne 1998). However, continuously varying the levels of heme or iron in the environment and tracking its effect on HupA production would allow us to see this switch in action. Testing the level at which the HupA system activates as a function of iron would be particularly useful. If these amounts of iron were found to differ, even at different numerical values than in the model, it would confirm the switch-like behavior of this gene regulatory network. As this hysteresis is robust to a number of parameter changes, this would generally confirm that this gene regulatory network functions as a switch. In this case, entering the bistable region from two different initial conditions could lead to two different results: if iron is initially high, HupA will not turn on; however, if iron is initially low and later increased, according to this model HupA should stay on, as in Figure 5. Validating this in a laboratory setting would confirm that heme and iron are the driving forces in this gene regulatory network and the existence of hysteresis. These switch like dynamics would also confirm that two dimers of HupR are used as an activating transcription factor, revealing a possible mechanism for this non-classical LysR type transcription factor. Further experimentation would also allow for the parameters of this model to be better tuned, enabling more exact predictions. Understanding how the levels of heme and iron within the blood govern this switch could offer important insights into understanding the initial establishment of infection as each individual bacterium makes the decision to activate this heme importation system.

This network relies on the presence of heme to activate the alternative iron seeking strategies. However, in the human body heme is primarily bound in the hemoglobin complex and stored inside red blood cells; it must be made available in the extracellular environment in order for the HupA uptake system to function. The previously mentioned toxin, VvhA, is excreted by *V. vulnificus *and causes red blood cells to lyse, increasing the level of heme in a bacteria’s immediate environment. This toxin is governed by a complex system of regulatory molecules, each of which allow the bacteria to produce differing amounts of VvhA in response to a variety of signals. These amounts of VvhA would affect the amount of external heme available for uptake, in contrast to this model where external heme concentration is taken to be a constant value. The interplay between VvhA induced hemolysis and HupA mediated heme importation offers a more complete picture of the initial establishment of *V. vulnificus *infection; a full understanding of this process could help determine more effective treatments.

**Supplementary information** Not applicable.

## Data Availability

We do not analyze or generate any datasets, because our work proceeds within a theoretical and mathematical approach.

## Appendix A A Note on Parameter Values

The values for parameters and rates in this model have largely not been reported on in the literature and would require further experimentation to determine. As such, nondimensional parameters used in the analysis here were chosen somewhat arbitrarily, though the system exhibits switch-like behavior and bistability of a large range of parameter values. This conservation of bistability can be seen in the two-parameter bifurcation graphs in Figures 6, 7, and 10; analogous plots can be found for $h_{ext}$ and any other model parameter of interest. A small value for leakage ($\epsilon$), relative to the other parameters in the model, arises due to the leaky expression of HupA, as seen in Figure 10(d). Larger relative values for $\alpha _3$ and $\kappa _2$ reflect volume difference between the cell interior and its exterior environment while the increase in $\alpha _{4-}$ is indicative of the bacteria’s metabolic need for iron. Increased values for $b_1$ and $b_2$ reflect the binding affinity of the various transcription factors to the shared *hupA / hupR* regulatory region, as discussed in further detail in Figure 6 and 7. The exact tuning of these pararmeters ensures that the model has the appropriate response of HupA depending on the levels of heme and iron in the environment.

**Table 1:** Parameter values used to generate figures 4, 5, 6, 7, 8, 9, and 10

Parameter	Value	Parameter	Value	Parameter	Value
$$ f_{ext}$$	0.1	$$\epsilon $$	0.01	$$f_T$$	1
$\kappa _1$	1	$\kappa _2$	10	$\kappa _3$	1
$\alpha _1$	1	$\alpha _2$	1	$\alpha _3$	10
$\alpha _4$	1	$\alpha _{4-}$	10		
$\beta _1$	4	$\beta _2$	2		

## Appendix B Code and Figures

The code used to generate Figs. 3-10 can be found at https://github.com/katlynch/HupA. Figure 3 generated in Matlab 2023a. Figures 4, 6, 7, 8, 9, and 10 generated in XPP AUTO (version 8.1) and plotted using Tsing Hao’s Matlab interface available at https://sites.pitt.edu/~phase/bard/bardware/xpp/xpp.html (using Matlab 2023a). Figures 5 generated in both XPP Auto (version 8.1) and Matlab (2023a).

## Appendix C Tables

**Table 2:** Nondimensionalization scaling for parameters and variables, with time scaling $\tau = a_{4} t$

Variables	Parameters		
$a = \frac{d_1}{a_1} A$	$\alpha _1 = \frac{d_1}{a_4}$	$\alpha _2 = \frac{d_2}{a_4}$	$\alpha _3 = \frac{a_3 a_4}{d_1 a_4}$
$r = \frac{d_2}{a_2} R$	$\alpha _4 = \frac{a_2 k_1}{d_2 k_{1-}}$	$\alpha _{4-} = \frac{d_4}{a_4}$	$f_T = \frac{k_{1-} F_T}{k_1}$
$h = \frac{a_4}{a_1} H$	$\kappa _1 = \frac{k_{1-}}{a_4}$	$\kappa _2 = \frac{k_2 k_1}{k_{1-} a_4}$	$\kappa _3 = \frac{k_3 k_{1-}}{k_1}$
$f^* = \frac{k_1}{k_{1-}}F^*$	$\beta _1 = \frac{b_1 a_2^{3n/2}}{d_2^{3n/2}}$	$\beta _2 = \frac{b_2 k_{1-}}{k_1 }$	$\epsilon = \frac{\epsilon }{a_1}$
$fe = \frac{k_1}{k_{1-}} Fe$	$h_{ext} = \frac{H_{ext}}{K_H}$	$fe_{ext} = \frac{Fe_{ext}}{K_F}$	
